# Is there variation between hospitals within each region in postoperative mortality for lung cancer surgery in France? A nationwide study from 2013 to 2020

**DOI:** 10.3389/fmed.2023.1110977

**Published:** 2023-03-14

**Authors:** Alain Bernard, Jonathan Cottenet, Pierre-Benoit Pages, Catherine Quantin

**Affiliations:** ^1^Department of Thoracic and Cardiovascular Surgery, Dijon University Hospital, Dijon, France; ^2^Service de Biostatistiques et d'Information Médicale (DIM), CHU Dijon Bourgogne, INSERM, Université de Bourgogne, CIC 1432, Module Épidémiologie Clinique, Dijon, France; ^3^Université Paris-Saclay, Université de Versailles Saint-Quentin-en-Yvelines (UVSQ), Inserm, Centre de recherche en Epidémiologie et Santé des Populations (CESP), Villejuif, France

**Keywords:** lung cancer surgery, standardized mortality rate, variation, region, quality of care

## Abstract

**Introduction:**

The practice of thoracic surgery for lung cancer is subject to authorization in France. We evaluated the performance of hospitals using 30-day post-operative mortality as a quality indicator, estimating its distribution within each region and measuring its variability between regions.

**Material and methods:**

All data for patients who underwent pulmonary resection for lung cancer in France (2013–2020) were collected from the national hospital administrative database. Thirty-day mortality was defined as any patient who died in hospital (including transferred patients) within the first 30 days after the operation and those who died later during the initial hospitalization. The Standardized Mortality ratio (SMR) was the smoothed, adjusted, hospital-specific mortality rate divided by the expected mortality. To describe the variation in hospital mortality between hospitals in each region, we used different commonly used indicators of variation such as coefficients of variation (CV), interquartile interval or range (IQR), extreme ratio, and systematic component of variance (SCV).

**Results:**

In 2013–2020, 87,232 patients underwent lung resection for cancer in France. The number of deaths was 2,537, a rate of 2.91%. The median SMR of 199 hospitals was 0.99 with an IQR of 0.86 to 1.18 and a CV of 0.25. Among the regions that had the most hospitals performing lung resections for cancer, the extreme ratio was >2, which means that the maximum value is twice as high as the minimum value. The SCV between hospitals was >10 for two of these regions, which is considered indicative of very high variation. For the other regions (with few hospitals performing lung resections for cancer), the variation between hospitals was lower. Globally, the variability between regions concerning the SMR was moderate, 6% of the variance was due to differences across regions. On the contrary, the hospital volume was significantly related to the SMR (*p* = 0.003) with a negative linear trend, whatever the region.

**Conclusion:**

This work shows significant differences in the practices of the various hospitals within regions. However, overall, the variability in the 30-day mortality rate between regions was moderate. Our findings raises questions regarding the regionalization of major surgical procedures in France.

## Introduction

The practice of lung cancer surgery in France has been the subject of several publications using the national hospital (PMSI) medico-administrative database (1–3). Although the number of hospitals performing this surgery was reduced following the implementation of the French National Cancer Plan (4), the number still remains high. The corollary of this situation is that many surgical teams have relatively low surgical activity (1, 2). The number of lung resections performed per year, called hospital volume, is one of the indicators influencing post-operative mortality (2, 5). Post-operative mortality is one of the quality indicators of lung cancer surgery, as recently demonstrated by Fernandez et al. (6).

Thoracic surgery for lung cancer (LC) is a surgical act that is subject to authorization in France, and this authorization is granted to hospitals by the regional authorities (4). There is yet to be an evaluation of the quality of care of hospitals in different regions in France for this type of surgery.

Our work consisted of evaluating the performance of hospitals using one quality indicator, 30-day post-operative mortality, and then estimating the distribution of this indicator within each region. Our secondary objective was to measure the variability of this indicator from one region to another.

## Materials and methods

### Data source and study population

All data for patients who underwent pulmonary resection for LC in France from January 2013 to December 2020 were collected from the national hospital administrative database. This database, called PMSI for “Programme de Médicalisation des Systèmes d'Information,” was inspired by the US Medicare system. The reliability and validity of PMSI data have already been assessed (7). Routinely collected medical information includes the principal diagnosis, secondary diagnoses and procedures performed. Diagnoses identified during the hospital stay are coded according to the International Classification of Diseases, tenth revision (ICD-10) (8). We selected patients for whom a diagnosis of primary lung cancer was coded as the principal discharge diagnosis (all codes C34). Procedures are coded according to the CCAM (Classification Commune des Actes Médicaux). For all patients, LC was confirmed by pathology analyses according to the 2004 World Health Organization classification of LC (7). Surgery-related variables included the surgical approach [thoracotomy, video assisted thoracic surgery (VATS) or robot-assisted surgery], the type of resection (limited resection, lobectomy, bi-lobectomy and pneumonectomy), bronchioplasty, and the extent of the pulmonary resection (to the chest wall, the left atrium, the carina, the diaphragm, and the superior vena cava).

Patient consent was not required. Ethics approval for use of this database was obtained from the French National Commission for Data protection (*Commission Nationale de l'Informatique et des Libertés*: No 1576793), and this study adhered to the tenets of the Declaration of Helsinsksi.

### Patient characteristics

Patient age and sex were included as baseline demographic characteristics. From the national administrative database, we included the following comorbidities: pulmonary disease (chronic bronchitis, emphysema), heart disease (coronary artery disease, cardiac arrhythmia, congestive heart failure, valvular heart disease, pulmonary artery hypertension, pulmonary embolism), peripheral vascular disease, liver disease, cerebrovascular events, neurological diseases (hemiplegia or paraplegia), renal disease, hematologic disease (leukemia, lymphoma), metabolic disease, anemia, other therapies (preoperative chemotherapy, steroids), and infectious disease. We also calculated the modified Charlson Comorbidity Index (CCI) as a marker of comorbidity (9).

### Region and hospital characteristics

Metropolitan France comprises 13 regions: Auvergne Rhones-Alpes (ARA), Bourgogne Franche-Comté (BFC), Bretagne (BRE), Centre Val-de-Loire (CVL), Corse (COR), Grand-Est (GE), Hauts-de-France (HdF), L'ile de France (IdF), Normandie (NOR), Nouvelle Aquitaine (NA), Occitanie (OC), Pays de Loire (PdL) and Provence Alpes Côte d'Azur (PACA). For each hospital within a region, we determined the number of times each type of pulmonary resection was performed from January 1, 2013, to December 31, 2020. Hospital volume was defined as the median number of procedures performed per year. For the purpose of the analysis, the hospital volume was represented as a continuous variable that was transformed into a logarithm.

### Outcome measurements

Thirty-day mortality for a patient was defined as the occurrence of death in hospital (including transferred patients) either within the first 30-days after the operation or later on during the initial hospitalization. In other words, in the calculation of 30-day in-hospital mortality, we included deaths that occurred during the surgical stay, as well as any death that occurred during a subsequent hospitalization within 30-days of admission for the initial surgery.

### Statistical analysis

To obtain a reliable measure of hospital quality, we used the hierarchical logistic regression model with “shrinkage” estimators. The adjusted Standardized Mortality ratio (SMR) was determined as the smoothed, adjusted, hospital-specific mortality rate divided by the expected mortality. The expected mortality was estimated from a fixed-effects component of hierarchical logistic model regression (10), using comorbidities, age, sex, modified CCI score, and the type of pulmonary resection (the approach and extent of resection) as adjustment factors. The hierarchical logistic regression model was developed using BUGS software (Bayesian Inference Using Gibbs Sampling, version 0.60; MRC Biostatistics Unit, Cambridge, United Kingdom). The credible 95% probability interval (PI) for each provider was then estimated.

In addition, we constructed funnel plots to determine outliers for 30-day mortality according to Spiegelhalter's methodology (11).

We used several methods to describe between-hospital differences in patient characteristics, the different procedures and the SMR of the hospitals within each region. Two groups of statistics of variation are commonly used: those that describe the distribution of rates, such as coefficients of variation (CV), interquartile, interval or range (IQR), extreme ratio, and those that use differences between expected and observed cases, such as the systematic component of variation (SCV) (12). The CV is the ratio of the standard deviation to the mean and measures how the data spreads around the average. The higher the coefficient of variation, the greater the level of dispersion around the mean. If the coefficient of variation is >1, it shows relatively high variability in the data sets. The IQR defines the interval or the range between the 1st and the 3rd quartile of the distribution, and measures how the data spreads around the average. The wider the range, the greater the level of dispersion around the mean. The extreme ratio corresponds to the ratio between the maximum and minimum values. An extreme ratio >2 implies that the maximum value is twice as high as the minimum value. Finally, SCV is the variation arising from the differences between the independent variable. A SCV >10 can be considered indicative of very high variation.

To estimate the potential influence of hospital volume on the SMR, we also studied the relationship between hospital volume and SMR by calculating a linear trend.

To estimate the variability of the SMR between regions, we used the intraclass correlation coefficient (ICC), which was calculated by fitting a multilevel regression model with a fixed coefficient for hospital volume and random intercept for the region.

The calculations for the hierarchical and multilevel logistics regression models were carried out using STATA 14 software (StataCorp, College Station, Tex), and for the full Bayesian analysis we used the R2jags module of R software (http://www.r-project.org).

## Results

**Figure 1:**
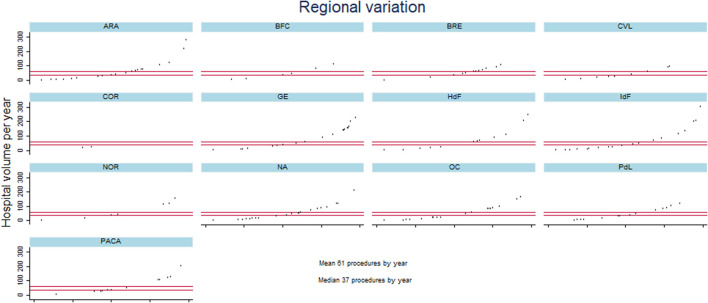
Description of hospital volume per year for the different hospitals in each French region from January 2013 to December 2020. Metropolitan France comprises 13 regions: ARA, Auvergne Rhones-Alpes; BFC, Bourgogne Franche-Comté; BRE, Bretagne; CVL, Centre Val-de-Loire; COR, Corse; GE, Grand-Est; HdF, Hauts-de-France; IdF, L'ile de France; NOR, Normandie; NA, Nouvelle Aquitaine; OC, Occitanie; PdL, Pays de Loire; and PACA, Provence Alpes Côte d'Azur. The X axis of each figure represents the different hospitals per region and the Y axis the hospital volume.

From 2013 to 2020, 87,232 patients underwent lung resection for cancer in France. The number of deaths was 2,537, resulting in a death rate of 2.91%. The variations in patient characteristics between the 199 hospitals in the 13 French regions is reported in Table 1. Regarding demographics, the median rate of female patients was 0.33, with an IQR of [0.28–0.37], and the median age was 65 years with IQR of [64–66 years] (Table 1). The pneumonectomy and bilobectomy rates varied highly across hospitals, resulting in a CV of 1.7 and 2.09, respectively (Table 1). Hospitals in the different regions had a VATS or robot-assisted surgery rate ranging from 0.075 to 0.47 with a CV of 0.89 (Table 1). The median hospital volume was 37, with an IQR of 9 to 87 procedures per year and a CV of 1.49. Figure 1 illustrates the high variability in hospital volume across the regions.

**Table 1:** Between hospital variation in the characteristics of patients undergoing lung cancer surgery in France, January 2013 to December 2020.

	**Mean**	**Median**	**IQR**	**cv**
**Gender**				
Female	0.31	0.33	0.28–0.37	0.53
Age (years)	65	65	64–66	0.09
**Comorbidities**				
Pulmonary disease	0.36	0.31	0.2–0.435	0.68
Heart disease	0.18	0.15	0.085–0.22	0.97
Peripheral vascular disease	0.087	0.07	0.02–0.13	1.18
Liver disease	0.007	0.01	0–0.01	1.47
Neurological disease	0.05	0.03	0.007–0.06	1.9
Metabolic disease	0.11	0.12	0.07–0.14	0.85
Renal disease	0.02	0.02	0–0.03	0.92
Anemia	0.15	0.10	0.045–0.19	1.22
Hematological disease	0.05	0.024	0.006–0.055	2.22
Infectious disease	0.005	0.0025	0–0.007	1.54
Other treatment	0.09	0.044	0.006–0.13	1.51
**Modified CCI score**				
1	0.098	0.07	0.017–0.143	1.32
2	0.113	0.085	0.05–0.13	1.28
≥3	0.39	0.38	0.24–0.5	0.62
**Type of pulmonary resection**				
Lobectomy	0.65	0.73	0.62–0.78	0.4
Bilobectomy	0.04	0.03	0.01–0.04	2.09
Pneumonectomy	0.075	0.06	0.03–0.086	1.7
**Surgery approach**				
VATS or Robot-assisted	0.31	0.24	0.075–0.47	0.89
Extended resection	0.13	0.06	0.014–0.13	1.52
30-day mortality rate	0.06	0.03	0.014–0.04	2.57

Cv, coefficient of variation (=standard deviation/mean); IQR, interquartile interval (1^*st*^ quartile – 3^*rd*^ quartile); CCI, Charlson Comorbidity Index. For the line regarding female gender, we observed that the average rate of women among the 199 hospitals was 31%, with a median of 33%. The IQR means that 50% of rate of women among these hospitals lie between 28 and 37%. Finally, the standard deviation of the rate of women is 53% the size of the mean (CV = 0.53).

### Standardized mortality rate

In order to calculate the SMR, the model used to obtain the expected 30-day mortality included 16 comorbidities, age, sex, modified CCI score, and the type of pulmonary resection (the approach and extent of resection). The reliability of 30-day mortality was 0.43 with 95% confidence interval (CI) of 0.32 to 0.53. The median SMR of 199 hospitals was 0.99 with an IQR of 0.86 to 1.18 and a CV of 0.25.

### Identification of quality-of-care outliers

**Figure 2:**
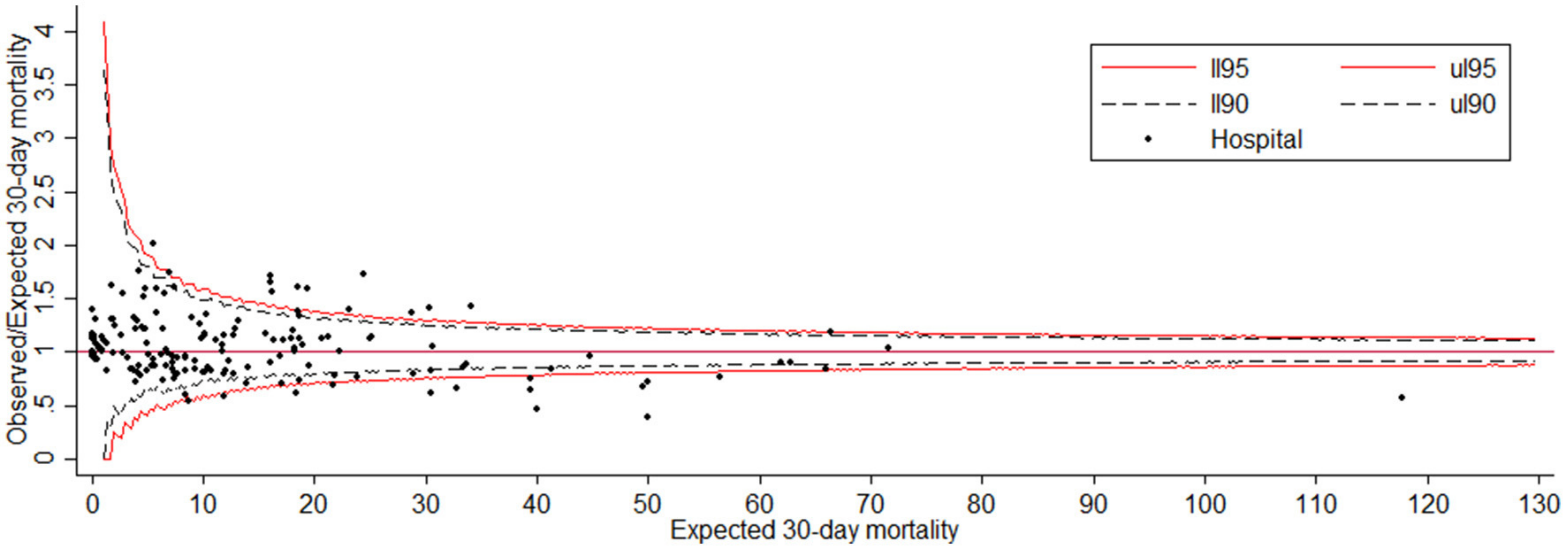
Funnel plot for the 30-day mortality with adjusted Observed/Expected ratio (SMR). The red line corresponds to the 95% control limit and the dash black line corresponds to the 90% control limit.

The funnel plot for the SMR describing hospital performance is displayed in Figure 2. It shows that out of 199 hospitals, 13 lie below the lower limit of the central 95% region, indicating performance that was better than expected. The thirteen hospitals that lie above the upper limit performed significantly worse than expected.

### Regional variation

Out of the 13 regions, four regions (COR, BFC, CVL and NOR) had less than ten hospitals performing lung resections for cancer, five regions (HdF, BRE, PdL, PACA and OC) had between 10 and 20 hospitals, and four regions (GE, NA, IdF and ARA) had between twenty and thirty hospitals.

The variation in the SMR between hospitals in different regions is reported in Table 2, which shows the following measures: mean, CV, extreme ratio, interquartile ratio and SCV. Amongst the regions with the highest number of hospitals performing lung resections for cancer, the extreme ratio (i.e., the ratio of the highest SMR to the lowest SMR) was >2. On the other hand, the SCV was >10 in four regions (ARA, IdF, NOR, and PdL), which can be considered indicative of very high variation in these regions. For the OC region, the SCV was 4.9, while the extreme ratio was 4.45. Some regions with few hospitals, such as BRE, CVL, NOR and PdL had a high variability with an extreme ratio around 2. For the other regions, the variation between hospitals was smaller.

**Table 2:** Between hospital variation in adjusted standardized mortality rates in each region of France.

**Region**	**Hospitals (*n*)**	**cv**	**Extreme ratio**	**Interquartile ratio**	**IQR**	**SCV**
Auvergne Rhone-Alpes	27	0.178	2.55	1.235	0.81–1.00	12.23
Bourgogne Franche-Comté	6	0.094	1.33	1.04	0.96–1.00	7.00
Bretagne	14	0.183	1.99	1.27	0.91–1.16	7.5
Centre-Val de Loire	9	0.223	2.41	1.18	0.96–1.13	1.85
Corse	2	0.453	1.94	1.94	0.82–1.60	1.13
Grand-Est	20	0.265	2.73	1.36	0.82–1.12	0.73
Hauts de France	13	0.190	1.80	1.35	0.75–1.01	1.18
Ile de France	26	0.330	3.86	1.42	0.82–1.17	18
Normandie	9	0.195	1.91	1.18	0.86–1.02	17
Nouvelle Aquitaine	23	0.244	2.83	1.55	0.83–1.29	2.52
Occitanie	19	0.270	4.45	1.3	0.94–1.21	4.90
Pays de Loire	15	0.275	2.44	1.62	0.96–1.55	11
Provence Alpes Cote d'Azur	16	0.220	2.00	1.33	0.99–1.32	2.80

cv, coefficient of variation (=standard deviation/mean), if greater than 1, it shows relatively high variability in the data sets; Extreme ratio (=Maximum/Minimum), greater than 2 implies that the maximum value is twice as high as the minimum value; Interquartile ratio, 75th percentile rate divided by the 25th percentile; IQR, interquartile interval (1^st^ quartile – 3^rd^ quartile); SCV, systematic component of variance, greater than 10 can be considered indicative of very high variation. For the line regarding Auvergne Rhone-Alpes, we observed that the standard deviation of SMR is 17.8% the size of the mean (CV = 0.178). Among the 27 hospitals, the maximum value of the SMR is more than twice as high as the minimum value of the SMR (extreme ratio = 2.55). The IQR means that 50% of the SMR among these hospitals lie between 0.81 and 1.00, knowing that the ratio between the 3rd quartile and the 1st quartile is 1.235. Finally, the SCV is 12.23.

**Figure 3:**
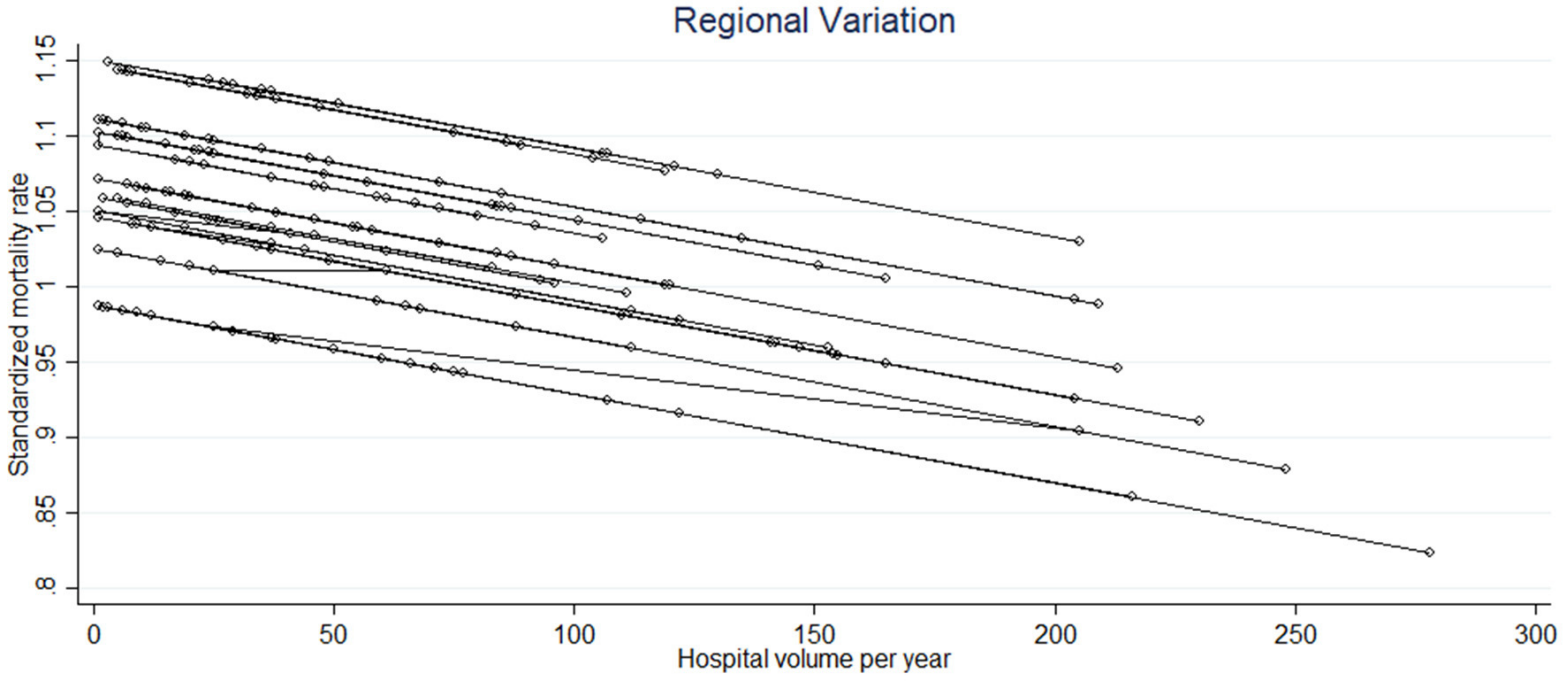
The significant linear relationship between the standardized mortality rate and the hospital volume of the different hospitals in each region (each circle corresponds to a hospital and each line to a region).

Figure 3 shows the relationship between the hospital volume (i.e., the median number of procedures performed per year) and SMR. The hospital volume was significantly related to the SMR (*p* = 0.003) with a negative linear trend, whatever the region (Figure 3). However, we noted a difference in the slope of the linear trend of the SMR between regions. The variability between regions was globally moderate with an ICC of 0.06 (95% CI: 0.014–0.23), indicating that 6% of the SMR variance is due to differences across regions. This is consistent with the value of IQRs observed from one region to another, seeing as the intervals overlap (Table 2). Statistically, this means that the SMRs are not different from one region to another.

## Discussion

To the best of our knowledge, this is the first study of this type to be carried out in France. The literature on the study of regional variations in surgical practice in hospitals is fairly poor (13–15). Our work underscores the fact that there is currently a large number of French hospitals performing resections for lung cancer. Surgical practice is dispersed and in-hospital mortality varies considerably from one hospital to another. The mean SMR was similar across regions, but with great variability between hospitals within regions. Nevertheless, the variability was more marked for certain regions, particularly those in which many hospitals perform the surgery.

Our work reveals that there is variability in the practices of the different hospitals as well as in the different regions. There are still many low-volume hospitals in France despite the required authorization. One of the characteristics of French health care is the dispersed supply of care, which is also seen in other European countries (16–19). However, the number of hospitals per region practicing this type of surgery is not in line with the needs of the population in regions such as in Auvergne Rhône-Alpes or Nouvelle Aquitaine.

To our knowledge, the variation in hospital comorbidities within regions has not yet been described in the literature. Our work reveals a considerable variation in the rate of certain comorbidities, such as peripheral vascular disease, liver disease, anemia and hematological disease. The coefficient of variation was 1.18, 1.47, 1.22, and 2.22, respectively. It is possible that the patients managed in the different hospitals are different in terms of comorbidities or that the coding for the associated PMSI diagnoses is not consistent from one hospital to another. Because of our data source, it is not possible to know which of these hypotheses is most likely to explain the differences observed.

The type of lung resection also varied from one hospital to another, particularly bilobectomies and pneumonectomies, and we cannot compare our results with the literature because lung cancer surgery has not been published using the same methodology as ours. Pneumonectomies are complex procedures more often performed by specialized teams in high volume units, which may explain the variability observed between hospitals.

The median 30-day mortality rate was 3%, which appears to be higher than in some European countries, which is consistent with a recent systematic review (20) comparing mortality following lung cancer resection in France with other European countries. The systematic review showed that France has a higher rate than some other European countries, including England, Denmark, Holland or Finland. Our work confirms the great variability in post-operative mortality between hospitals, as shown by the interquartile values and the coefficient of variation. It is a complex issue to understand why France (20) has a higher rate of post-operative mortality than other European countries.

The fact that volume of hospital activity is significantly related to excess mortality (21–25) has been demonstrated many times in the literature. Countries in which fewer centers offer thoracic surgery, such as in the UK, have a lower overall post-operative mortality rate than France. On average, their centers also have a higher volume of activity than French centers (16–19), and an interesting English study showed that high-volume centers had better survival after lung cancer surgery (23). The literature unanimously illustrates that high-volume centers have better results in terms of mortality, confirmed by our study, whatever the French region. Obviously, hospital volume is not the only component of quality of care that can have a role in hospital mortality, and we know that the nurse to patient staffing ratio or the processes in the different units could have an important role. For example, in a study by Aiken et al. (26) in nine European countries, the authors showed that an increase of one patient per nurse is associated with a 7% increase in the risk of a surgical patient dying within 30-days of admission. Further studies are needed to assess these additional factors, but we unfortunately do not have access to such data in the PMSI database.

Our work provides an estimation of the variability in post-operative mortality between regions, a subject for which the literature is scarce (27). We have shown that the difference in 30-day mortality between regions is ultimately moderate, and the most striking variability was observed between hospitals within regions. Our findings raise questions regarding current regionalization policies for major surgical procedures such as lung cancer surgery. Some countries have moved toward the centralization of complex surgeries in selected hospitals in an attempt to group together the technical skills needed to improve quality of care (28, 29). This idea seems attractive, especially if we want to be able to measure hospital performance reliably. Compared with other countries, France offers dispersed care for major interventions like thoracic surgery or pancreatic surgery, which could be one of the reasons that the death rate is higher in France than in other countries (20).

The differences in the 30-day mortality of hospitals in France could also be explained by the lack of quality measurement, in contrast to certain European countries (16–19). European surgical teams that undertake regular assessments of their practice are informed of their level of performance and can thus implement actions to improve their results.

We recognize that our study has some limitations. In particular, the choice of mortality as the key outcome is a limitation because we did not assess the overall quality of care but only one indicator, the 30-day mortality rate, which is only one of several potential indicators. Other indicators, for instance post-operative complications after lung resection, such as pneumopathy or atelectasis, which are good quality indicators, cannot be obtained through the PMSI. We only had access to in-hospital mortality data, but it is nevertheless a relevant indicator for this type of surgery. Another limitation concerns the TNM stage, which is not recorded in the PMSI, but is a factor that can influence mortality. The final limitation concerns the risk of underestimating certain comorbidities because of the quality of the coding. Indeed, coding practices may vary among institutions since the people who perform the coding of diagnoses can be clinicians or information system technicians. Nevertheless, the quality of coding is checked in a standardized way by medical information professionals in each hospital in order to correct diagnoses (internal quality assessment), and the level of re-cording of co-morbidities has increased significantly in recent years, following its impact on the tariff of hospital stays. Because of the impact on hospital budget allocation, it has been shown that hospital claims data are becoming more accurate. In addition, a national external quality assessment program has been implemented to verify the quality of discharge abstracts in each hospital.

In conclusion, this work shows significant differences in the practices of the various hospitals within each French region. However, between regions, there were only moderate variations in the 30-day mortality rate. These results should encourage surgical teams in France to implement quality of care measures based on the analysis of outcomes. Finally, our findings call into question the current policies relative to the regionalization of major surgical procedures in France.

## Data availability statement

The datasets presented in this article are not readily available because the use of these data by our department was approved by the National Committee for data protection. We are not allowed to transmit these data. PMSI data are available for researchers who meet the criteria for access to these French confidential data (this access is submitted to the approval of the National Committee for data protection) from the national agency for the management of hospitalization (ATIH-Agence technique de l'information sur l'hospitalisation). Requests to access the datasets should be directed to Agence technique de l'information sur l'hospitalisation 117 boulevard Marius Vivier Merle 69329 Lyon Cedex 03.

## Author contributions

AB was involved in the conception and design of the study, in charge of the analysis, involved in the interpretation, wrote the first draft, and approved the final version. JC was responsible for the data collection, accessed and verified the data, involved in the interpretation, critically reviewed the first draft, and approved the final version. P-BP was involved in the conception and design of the study, involved in the interpretation, critically reviewed the first draft, and approved the final version. CQ was the coordinator of the study, responsible for the data collection, accessed and verified the data, involved in the interpretation, and critically reviewed the first draft. All authors approved the final version.
